# Electromagnetic Tunneling and Resonances in Pseudochiral Omega Slabs

**DOI:** 10.1038/srep41961

**Published:** 2017-02-06

**Authors:** Faroq Razzaz, Majeed A. S. Alkanhal

**Affiliations:** 1 King Saud University, Electrical Engineering, Riyadh, 11421, Saudi Arabia

## Abstract

This paper presents theoretical investigation of the electromagnetic wave tunneling and anomalous transmission around the trapped modes in a pseudochiral omega slab. The dispersion relation, the conditions of the trapped modes, and the evanescent wave coupling and tunneling in two different reciprocal pseudochiral omega slab structures are derived. The Berreman’s matrix method is applied to obtain the transmission coefficients across the pseudochiral omega slab. When the structure is perturbed, a resonance phenomenon is detected around the trapped modes. This resonance results in transmission anomalies (total transmission and total reflection) and dramatic field amplifications around the trapped modes. The number of the discrete trapped modes and then the resonance frequencies are prescribed by the parameters of the pseudochiral omega slab such as the value of the omega parameter and its orientation and the slab thickness.

Material technology is succeeding towards creating new materials with specific electromagnetic (EM) and optical properties which do not exist in natural materials. Pseudochiral omega material is a motivating complex medium which reveals great potential for applications in microwave, millimeter and terahertz devices. The pseudochiral omega medium can be obtained by doping a host dielectric medium with Ω-shaped planar conducting microstructures. This material was presented for the first time in 1992 by Saadoun and Engheta^1^. Transverse electric (TE) and transverse magnetic (TM) electromagnetic wave polarizations are decoupled and are perpendicular to each other in this medium. Hence, the pseudochiral omega medium can be classified as a nonchiral medium^2^.

Several authors carried researches relevant to electromagnetic and optical wave behavior in pseudochiral omega media. Plane wave propagation in uniaxial pseudochiral omega media is presented with emphasizing some properties that are useful in designing EM devices^3^. Reflection and transmission characteristics of grounded and ungrounded uniaxial chiro-omega slabs is affected when the orientation of the omega elements is changing^4^. The characteristics of the radiation pattern of a pseudochiral point-source antenna, which consists of an elemental antenna embedded in a grounded pseudochiral substrate, showing the effects of frequency, slab’s thickness, constitutive parameters, and location of the source have been, also, examined^5^. The constitutive parameters of a pseudochiral omega medium can be extracted from the reflection and transmission coefficients in a rectangular waveguide using some TE/TM modes^6^. In another study, a rectangular waveguide with a pseudochiral omega slab with different localizations is used to predict the values of the slab constitutive parameters and to study the scattering characteristics of the omega slab in the guide^7^. The omega parameter has noticeable effects on the propagation constant of different hybrid modes in parallel plate^8^, circular^9^, and partially filled^10^ pseudochiral omega waveguides. Pseudochiral omega media can be used in the design of non-radiative dielectric directional couplers^11^. Asymmetric pseudochiral slab waveguide, where both the film and the substrate are made of pseudochiral Ω media, can support semi-leaky modes radiating energy into the substrate with Ω parameter exceeding a certain value^12^. A planar transmission line filled with a homogeneously uniaxial pseudochiral omega medium can support both TE and TM modes separately^13^. Under definite conditions, a periodically loaded transmission line can be considered as an effective omega media^14^. It is also shown that when a linearly polarized wave incident from vacuum onto a pseudochiral omega medium, the transmitted waves are elliptically polarized, while the reflected waves continue as linearly polarized^15^.

This work studies the behavior of the electromagnetic propagating and evanescent waves in a pseudochiral omega slab emphasizing the evanescent wave coupling and tunneling across the surfaces of the slab. The formation of discrete trapped modes and the conceivable resonances with the associated field amplifications in the pseudochiral omega slab are, correspondingly, investigated. The Berreman’s matrix method is developed to derive the transmission coefficients in both the non-resonant and the resonant states. Additionally, the instantaneous tangential field components are presented to display the field amplifications at the resonant frequencies. The effects of the omega elements and their orientation on the band where the trapped modes are embedded, the number of the discrete trapped modes, and consequently on the resonances are reconnoitered as well. Such an EM behavior in synthesized material is expected to have a remarkable influence on diagnosing and characterizing such materials and consequently to have several implications in the design of imminent electromagnetic and optical devices.

## Theoretical Formulation and Analysis

**Figure 1:**
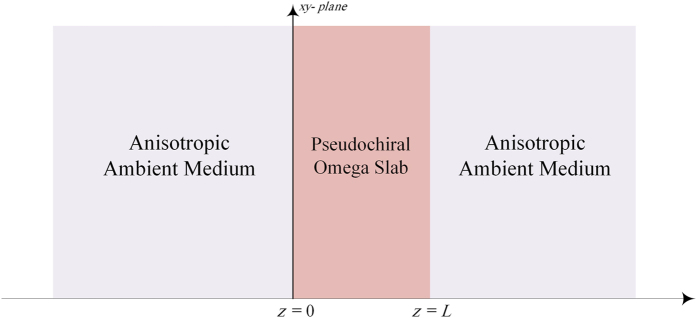
Geometry of the problem.

To investigate the behavior of the evanescent and propagating electromagnetic waves around the pseudochiral omega slab, consider the physical model shown in Fig. 1, where a reciprocal pseudochiral omega slab is placed in an anisotropic ambient medium. The ambient anisotropic media supports both propagating and evanescent modes at the same parallel wavevector. The Berreman matrix method is used in this work to explore the proposed physical problem. This method is based on reducing the Maxwell equations to four ordinary differential equations in each medium. Brief background about Berreman matrix method is presented in Appendix A.

### Fields in the anisotropic ambient medium

The general constitutive relations that characterize the anisotropic media are given in Eq. (B.1) in the appendices. Additionally, the anisotropic ambient medium is characterized by the properties given in Eq. (B.3). These properties provide two real and two imaginary *z*-directional wavenumbers corresponding to propagating and evanescent modes. These wavenumbers are the eigenvalues of the 4 × 4 matrix *iJA* in the medium and the wave modes in that medium are represented by the associated eigenvectors (see Appendix B for more details). At *k*_*y*_ = 0, the *z*-directional propagating and evanescent wavenumbers in the anisotropic ambient medium are given, respectively, by


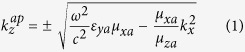



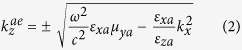


where the superscripts ‘*ap*’ and ‘*ae*’ refer to propagating and evanescent modes in the ambient medium respectively. The corresponding tangential field components (eigenvectors) associated with these wavenumbers are given by


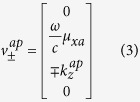



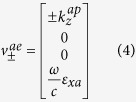


The propagating *z*-directional wavenumber 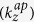 should be real, while the evanescent *z*-directional wavenumber 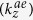 should be imaginary. Therefore, for anisotropic ambient medium that support these two modes instantaneously, the elements of the permittivity tensor 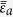 and permeability tensor 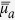 must satisfy the following conditions:


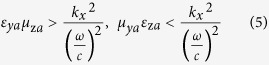


which implies that *ε*_*ya*_*μ*_z*a*_ > *μ*_*ya*_*ε*_z*a*_.

### Fields in the Pseudochiral Omega (Ω) Slab

**Figure 2:**
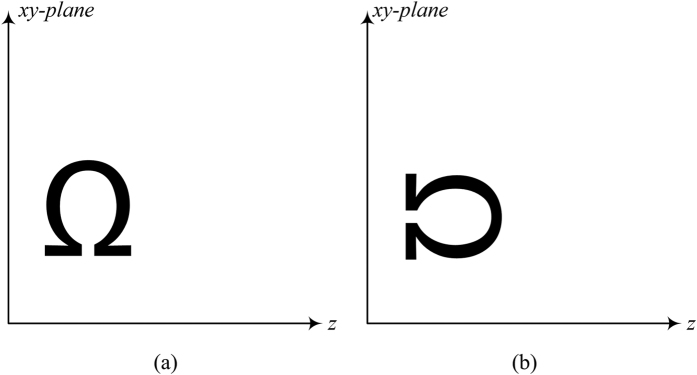
Orientation of the omega particles of the pseudochiral omega medium. (**a**) Ω_*yz*_ structure. (**b**) Ω_*xy*_ structure.

The constitutive relations that define the pseudochiral omega slab are given in Appendix C. Additionally, two different configurations for the pseudochiral omega slab are considered as shown in Fig. 2. The properties of these two configurations are given in Eq. (C.1) and Eq. (C.3) (see Appendix C for more details).

For the Ω_*yz*_ structure, the propagating and evanescent modes of the *z*-directional wavenumbers are given respectively by


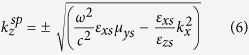



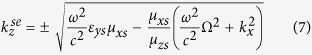


where the superscripts ‘*sp*’ and ‘*se*’ refer to the propagating and evanescent modes in the pseudochiral omega slab medium respectively. The propagating *z*-directional wavenumber 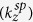 should be real, while the evanescent *z*-directional wavenumber 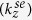 should be imaginary. Therefore, for Ω_*yz*_ medium that support these two modes instantaneously, the properties of the mediummust satisfy the following conditions:


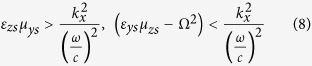


which implies that *ε*_*zs*_*μ*_*ys*_ > (*ε*_*ys*_*μ*_*z*_ − Ω^2^).

The corresponding tangential eigenfield components associated with these wavenumbers are also given by


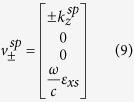



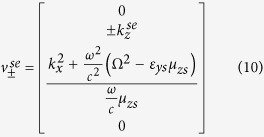


The trapped modes in the slab are characterized by 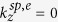 (no *z*-directional propagation), and their frequencies belong to the continuous interval *I* bounded by the dispersion curves of the propagating and evanescent waves of the slab given by Eqs (6) and (7), respectively, i.e.


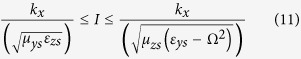


Evanescent wave coupling arises when the incident frequencies belong to the continuous spectrum interval *I* and the tangential field components of the anisotropic ambient evanescent modes match those of the propagating modes in the pseudochiral omega slab. If the normalized parallel wavevector is perturbed, resonances around the trapped modes will occur. Conversely, the propagating waves in the anisotropic ambient medium with frequencies that belong to the band *I* can be tunneled to the other side of the slab by means of evanescent waves across the pseudochiral slab. This phenomenon is similar to the tunneling of particles in quantum mechanics. Therefore, the construction of the trapped modes in the slab can be determined by matching the evanescent fields in the ambient medium with the propagating fields in the slab.

The fields in the anisotropic ambient media (*z* < 0 and *z* > *L*) and in the pseudochiral omega slab (0 < *z* < *L*) are given by


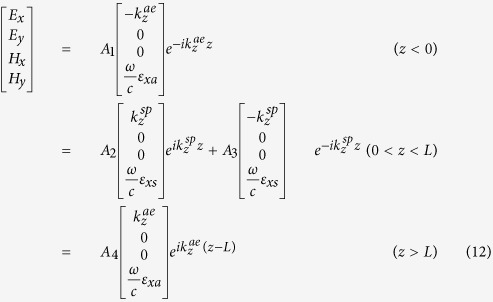


where *A*_1_, *A*_2_, *A*_3_ and *A*_4_ are constants to be determined by applying the appropriate boundary conditions. The continuity of the tangential field components at the interfaces (*z* =0 , *z* = *L*) yields the following:


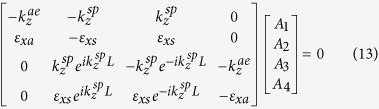


For the above matrix to have a non-trivial solution, its determinant must be equal to zero, then the condition of the trapped modes is given by


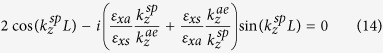


In the same manner, the propagating and evanescent modes of the *z*-directional wavenumbers for the *Ω*_*xy*_ structure, are given, respectively, by


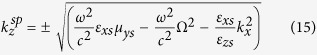



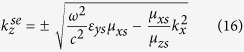


The corresponding tangential eigenfield components associated with these wavenumbers are also given by


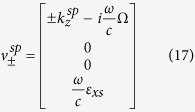



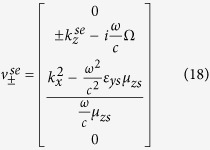


Additionally, the properties of the medium must satisfy the following conditions


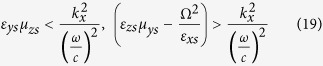


which implies that 
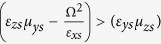
.

Moreover, the trapped modes condition in this case is given by


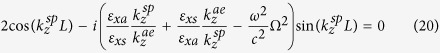


These trapped modes are embedded in the interval *I* given by


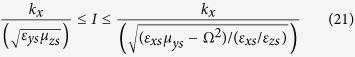


When an electromagnetic field is incident on the pseudochiral omega slab, part of this field will be transmitted to the other side and the other part of the field will be reflected. If the incident field hits the pseudochiral omega slab at *z* = 0, the resulting field outside the slab will be as follows:


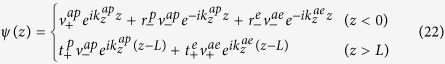


where, 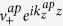 is the incident field, 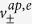 are the eigenvectors associated with the eigenvalues (*z*-directional wavenumbers) 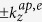 in the anisotropic ambient medium, and 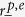 and 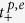 are the amplitudes of the reflected and transmitted fields, respectively. The tangential field components at the interfaces of the pseudochiral omega slab are continuous, so the field inside the pseudochiral omega slab can be found using the transfer matrix method^16^ as


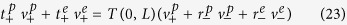


where 
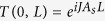
 is the transfer matrix of the pseudochiral omega slab. The transmission and reflection coefficients can be determined directly from Eq. (23).

## Results and Discussion

To explore the behavior of the propagating and the evanescent electromagnetic wave coupling and tunneling through the pseudochiral omega slab, the preceding derived analytical formulations are used to examine the dispersion relations, trapped modes condition, transmission coefficients in both the non-resonance and resonance cases, and the transmission anomalies at the resonance frequencies for the two omega structures.

Consider the anisotropic ambient medium characterized by the following properties


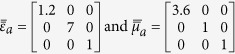


The permittivity and permeability tensors of the pseudochiral omega slab for the two structures are


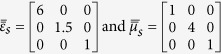


**Figure 3:**
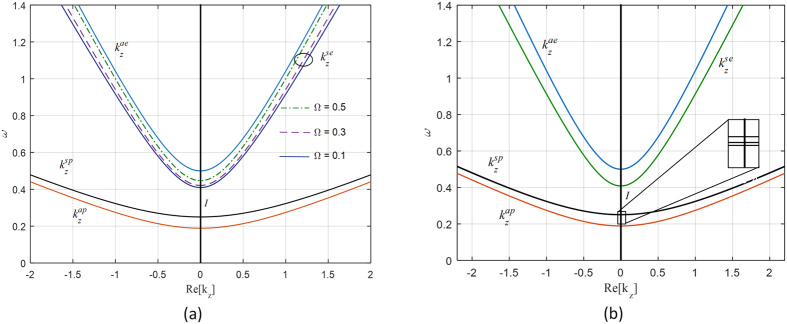
The dispersion relation for (**a**) Ω_*yz*_ structure, (**b**) Ω_*xy*_ structure. The blue and red lines are for the ambient medium propagating and evanescent *z*-directional wavenumber respectively. The black line is the propagating *z*-directional wavenumber of the pseudochiral omega slab. The green lines in (**a**) represent the evanescent *z*-directional wavenumber of the pseudochiral omega slab for three different values of the omega parameter. The interval *I* is bounded by the *z*-directional wavenumbers of the pseudochiral omega slab.

The dispersion relations of the *Ω*_*yz*_ and the *Ω*_*xy*_ structures are shown in Fig. 3(a) and in Fig. 3(b) respectively. For the *Ω*_*yz*_ structure, the continuous interval *I* where the trapped modes are embedded is widened as the value of the omega parameter is increased. The interval *I* against three different values of the omega parameter is listed in Table 1. In the *Ω*_*xy*_ structure, the continuous interval *I* is slightly decreases as the value of the omega parameter increases as listed in Table 2.

**Figure 4:**
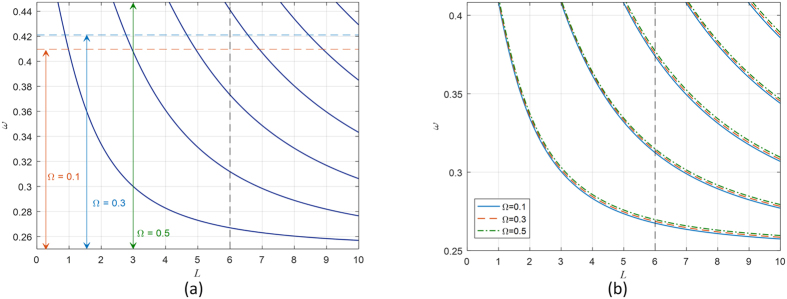
Trapped modes conditions for (**a**) the *Ω*_*yz*_ structure. (**b**) the *Ω*_*xy*_ structure.

Figure 4(a,b) shows the trapped modes embedded in the continuous interval *I* for the *Ω*_*yz*_ and *Ω*_*xy*_structures, respectively. It is clearly shown that the omega parameter has no effect on the frequencies of the trapped modes in the *Ω*_*yz*_ structure. However, as a result of expanding the continuous interval *I*, the number of the trapped modes may also be increased.

For example, when the thickness of the pseudochiral slab is equal to 6 unit length, the number of the trapped modes is three, when Ω = 0.1, 0.3, but there are four trapped modes when Ω = 0.5. In the *Ω*_*xy*_ structure, there is no change in the number of the trapped modes. However, a slight change in the interval *I* is introduced and the frequencies of the trapped modes are slightly altered as a results of the increasing omega parameter as shown in Fig. 4b. This can be inferred, also, from eqs (20) and (25).

**Figure 5:**
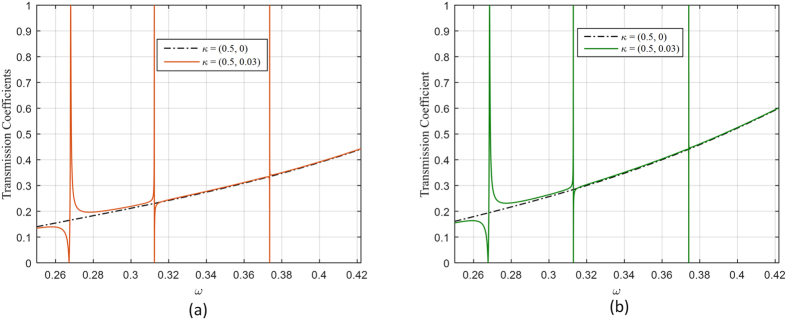
Transmission coefficient for (**a**) the *Ω*_*yz*_ structure, (**b**) the *Ω*_*xy*_ structure.

**Figure 6:**
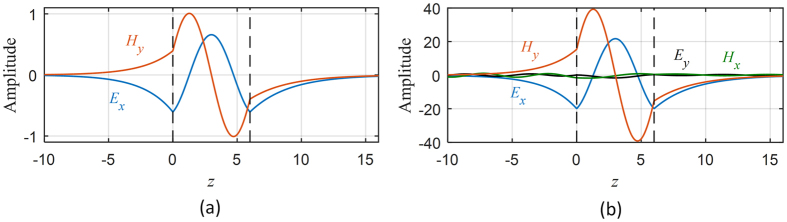
Instantaneous transverse fields. (**a**) At trapped mode frequency (*ω* = 0.266941, Ω = 0.3). (**b**) At resonance (*ω* = 0.267354, Ω = 0.3, *and κ* = (0.5, 0.02)).

**Figure 7:**
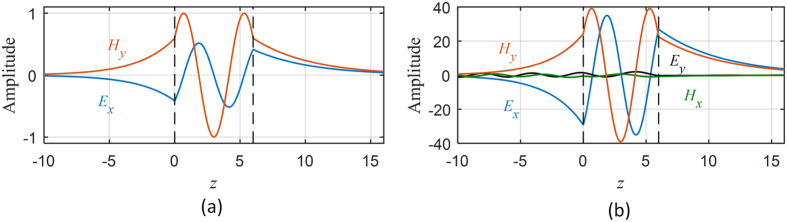
Instantaneous transverse fields. (**a**) At trapped mode frequency (*ω* = 0.311802, Ω = 0.3). (**b**) At resonance (*ω* = 0.312052, Ω = 0.3, and *κ* = (0.5, 0.02)).

The transmission coefficients for the two omega structures are shown in Fig. 5. When the parallel wavevector is set to *κ* = (0.5, 0), the pseudochiral omega slab admits three trapped modes (Ω = 0.3). Moreover, if the parallel wavevector is perturbed, resonances will occur around theses trapped modes. The resonances around the trapped modes for the parallel wavevector *κ* = (0.5, 0.03) are characterized by sharp transmission anomalies and field amplifications. The instantaneous field components at some trapped modes for a non-resonance state and for a resonance state are depicted in Fig. 6 and in Fig. 7 for the Ω_*yz*_ and *Ω*_*xy*_ structures respectively. The instantaneous fields display the coupling between evanescent waves in the anisotropic ambient medium to the propagating fields in the pseudochiral omega slab.

**Figure 8:**
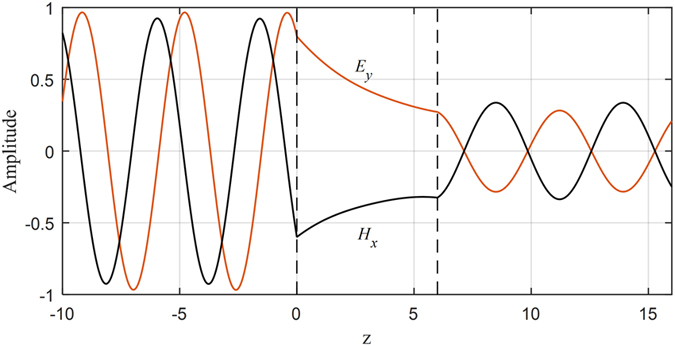
Tunneling of the fields across the slab, *ω* = 0.35 and  Ω = 0.3.

For any frequency that belongs to the continuum frequency b and *I*, propagating fields in the anisotropic ambient medium can be tunneled to the other side of the slab by the means of the evanescent fields in the pseudochiral slab as shown in Fig. 8.

## Conclusions

This paper presents analytical investigation of the electromagnetic wave coupling, tunneling, and resonances in a pseudochiral omega slab. It focuses on the characterization of the evanescent wave behavior and the physical conditions of the evanescent wave coupling and tunneling through the pseudochiral omega slab. The number and the frequencies of the discrete trapped modes possible in the pseudochiral omega slab are determined. The value and orientation of the omega parameter have an apparent effect on the trapped mode frequencies and on the width of the continuous interval where these trapped modes are embedded. Field resonances with sharp transmission/reflection anomalies and with intense field amplification in the pseudochiral omega slab are verified to arise when perturbing the parallel wavevector of the incident field around the trapped mode frequencies. Electromagnetic wave tunneling through evanescent waves in the slab is confirmed for incident waves with frequencies belong to the continuum frequency interval of the pseudochiral slab.

## Additional Information

**How to cite this article**: Razzaz, F. and Alkanhal, M. A. S. Electromagnetic Tunneling and Resonances in Pseudochiral Omega Slabs. *Sci. Rep.* **7**, 41961; doi: 10.1038/srep41961 (2017).

**Publisher's note:** Springer Nature remains neutral with regard to jurisdictional claims in published maps and institutional affiliations.

## Supplementary Material

Supplementary Appendices

## Figures and Tables

**Table 1:** Interval *I* for different values of the omega parameter for the Ω_*yz*_ structure.

Ω	Interval *I*
0.1	[0.25, 0.409615]
0.3	[0.25, 0.421075]
0.5	[0.25, 0.447213]

**Table 2:** Interval *I* for different values of the omega parameter for the Ω_*xy*_ structure.

Ω	Interval *I*
0.1	[0.250052, 0.408248]
0.3	[0.250470, 0.408248]
0.5	[0.251312, 0.408248]

## References

[b1] Saadoun M. M. & Engheta N. A reciprocal phase shifter using novel pseudochiral or Ω medium. Microwave and Optical Technology Letters 5, 184–188 (1992).

[b2] Topa A. L., Paiva C. R. & Barbosa A. M. Electromagnetic wave propagation in omega waveguides: Discrete complex modes and application to a ridge waveguide. Progress In Electromagnetics Research 49, 309–331 (2004).

[b3] Lindell I. V., Tretyakov S. A. & Viitanen A. J. Plane‐wave propagation in a uniaxial chiro‐omega medium. Microwave and Optical Technology Letters 6, 517–520 (1993).

[b4] Wenyan Y. & Wengbing W. Reflection and transmission for planar structures of uniaxial chiro‐omega media. Microwave and Optical Technology Letters 7, 475–478 (1994).

[b5] Toscano A. & Vegni L. Novel characteristics of radiation patterns of a pseudochiral point‐source antenna. Microwave and Optical Technology Letters 7, 247–250 (1994).

[b6] Norgren M. & He S. Reconstruction of the Constitutive Parameters for an O Material in a Rectangular Waveguide. IEEE transactions on microwave theory and techniques 43, 1315–1321 (1995).

[b7] Dorko K. & Mazur J. Characterization of rectangular waveguide with a pseudochiral/spl omega/slab. IEEE microwave and wireless components letters 12, 482–484 (2002).

[b8] Wenyan Y., Wei W. & Xiaowei S. Mode characteristics in a lossy parallel‐plate uniaxial chiro‐omega waveguide. Microwave and Optical Technology Letters 7, 868–870 (1994).

[b9] Wenyan Y., Wei W. & Wenbing W. Mode characteristics in a circular uniaxial chiro-omega waveguide. Electronics Letters 30, 1072–1074 (1994).

[b10] Mazur J. & Pietrzak D. Field displacement phenomenon in a rectangular waveguide containing a thin plate of Ω medium. IEEE microwave and guided wave letters 6, 34–36 (1996).

[b11] Topa A., Paiva C. & Barbosa A. In Microwave Conference, Asia-Pacific. 1238–1241 (IEEE) 2000.

[b12] Topa A. L., Paiva C. R. & Barbosa A. M. In Advances in Electromagnetics of Complex Media and Metamaterials 291–305 (Springer, 2002).

[b13] Hatefi-Ardakani H. & Rashed-Mohassel J. Study of mode propagation in pseudochiral transmission lines. Progress In Electromagnetics Research M 10, 39–47 (2009).

[b14] Vehmas J., Hrabar S. & Tretyakov S. Omega transmission lines with applications to effective medium models of metamaterials. Journal of applied physics 115, 134905 (2014).

[b15] Chern R.-L. & Chang P.-H. Wave propagation in pseudochiral media: generalized Fresnel equations. JOSA B 30, 552–558 (2013).

[b16] Berreman D. W. Optics in stratified and anisotropic media: 4 × 4-matrix formulation. Josa 62, 502–510 (1972).

