# Supplementary material for: Electromagnetic Tunneling and Resonances in Pseudochiral Omega Slabs
Source: Sci Rep. 2017 Feb 6;7:41961. doi: 10.1038/srep41961 (PMC5292747; doi:10.1038/srep41961)
Supplement: Supplementary Appendices [file srep41961-s1.pdf]

Electromagnetic Tunneling and Resonances in Pseudochiral Omega Slabs

Faroq Razzaz and Majeed A. S. Alkanhal

Appendices

Appendix A

The general constitutive relations that represent the pseudochiral omega medium are given by

$$\begin{aligned} \bar{D} &= \bar{\bar{\varepsilon}}\bar{E} + \bar{\bar{\xi}}\bar{H} \\ \bar{B} &= \bar{\bar{\zeta}}\bar{E} + \bar{\bar{\mu}}\bar{H} \end{aligned} \tag{A.1}$$

Where $\bar{\bar{\varepsilon}}$ is the 3×3 permittivity tensor, $\bar{\bar{\mu}}$ is the 3×3 permeability tensor, and $\bar{\bar{\xi}}$ and $\bar{\bar{\zeta}}$ are the magnetoelectric tensors.

Consider a general obliquely incident plane wave with $e^{-i\omega t}$ time dependency. The fields in the medium are defined as:

$$\mathbf{E}(x,y,z;t) = \mathbf{E}_0 e^{i(k_x x + k_y y + k_z z - \omega t)} = \begin{bmatrix} E_x(z) \\ E_y(z) \\ E_z(z) \end{bmatrix} e^{i(k_x x + k_y y - \omega t)} \tag{A.2}$$

$$\mathbf{H}(x,y,z;t) = \mathbf{H}_0 e^{i(k_x x + k_y y + k_z z - \omega t)} = \begin{bmatrix} H_x(z) \\ H_y(z) \\ H_z(z) \end{bmatrix} e^{i(k_x x + k_y y - \omega t)} \tag{A.3}$$

Maxwell's equations can be rewritten in matrix form as

$$\begin{bmatrix} 0 & -\nabla\times \\ \nabla\times & 0 \end{bmatrix} \begin{bmatrix} \bar{E} \\ \bar{H} \end{bmatrix} = i\frac{\omega}{c} \begin{bmatrix} \bar{D} \\ \bar{B} \end{bmatrix} \tag{A.4}$$

where $\nabla\times$ represents the curl operator on the fields. Considering the media given by Eq. (A.1) yields

$$\begin{bmatrix} 0 & -\nabla\times \\ \nabla\times & 0 \end{bmatrix} \begin{bmatrix} \bar{E} \\ \bar{H} \end{bmatrix} = i\frac{\omega}{c} \begin{bmatrix} \bar{\bar{\varepsilon}} & \bar{\bar{\xi}} \\ \bar{\bar{\zeta}} & \bar{\bar{\mu}} \end{bmatrix} \begin{bmatrix} \bar{E} \\ \bar{H} \end{bmatrix} \tag{A.5}$$

Then, Maxwell's equations can be reduced to four ordinary differential equations in terms of the tangential components of the electric and magnetic fields[16].

$$\frac{d\Psi(z)}{dz} = iJA\Psi(z) \tag{A.6}$$

where:

$$\Psi(z)=\begin{bmatrix} E_x(z) \\ E_y(z) \\ H_x(z) \\ H_y(z) \end{bmatrix},\ J=\begin{bmatrix} 0 & 0 & 0 & 1 \\ 0 & 0 & -1 & 0 \\ 0 & -1 & 0 & 0 \\ 1 & 0 & 0 & 0 \end{bmatrix}$$

Additionally, A is the 4×4 matrix that depends on the properties of the medium, the parallel wavevector, and the incident angular frequency. The general solution of Eq. (A.6) is given by[16]

$$\psi(z) = e^{iJAz}\psi(z_0) = Ve^{\lambda z}V^{-1}\psi(z_0) \tag{A.7}$$

where V and λ are the eigenvectors and the eigenvalues of the matrix iJA, respectively.

Appendix B

For anisotropic media, the constitutive relations shown in Eq. (A.1) reduce to the following

$$\begin{aligned} \bar{D} &= \bar{\bar{\varepsilon}}\bar{E} \\ \bar{B} &= \bar{\bar{\mu}}\bar{H} \end{aligned} \tag{B.1}$$

where $\bar{\bar{\varepsilon}}$ is the 3×3 permittivity tensor and $\bar{\bar{\mu}}$ is the 3×3 permeability tensor given by:

$$\bar{\bar{\varepsilon}}=\begin{bmatrix} \varepsilon_{xx} & \varepsilon_{xy} & \varepsilon_{xz} \\ \varepsilon_{yx} & \varepsilon_{yy} & \varepsilon_{yz} \\ \varepsilon_{zx} & \varepsilon_{zy} & \varepsilon_{zz} \end{bmatrix},\ \text{and}\ \bar{\bar{\mu}}=\begin{bmatrix} \mu_{xx} & \mu_{xy} & \mu_{xz} \\ \mu_{yx} & \mu_{yy} & \mu_{yz} \\ \mu_{zx} & \mu_{zy} & \mu_{zz} \end{bmatrix}$$

Referring to Eq. (A.6), the 4×4 matrix A depends on the properties of the medium ($\bar{\bar{\varepsilon}}$ and $\bar{\bar{\mu}}$), the tangential wavevector components, and the frequency of the incident wave. It is given by

$$A=\begin{bmatrix} a_{11} & a_{12} \\ a_{21} & a_{22} \end{bmatrix} \tag{B.2}$$

The elements of the above matrix A are given for a general anisotropic medium as

$$a_{11}=\begin{bmatrix} \frac{\omega}{c}\varepsilon_{xx}-\frac{c}{\omega}\frac{k_y^2}{\mu_{zz}}-\frac{\omega}{c}\frac{\varepsilon_{xz}\varepsilon_{zx}}{\varepsilon_{zz}} & \frac{\omega}{c}\varepsilon_{xy}+\frac{c}{\omega}\frac{k_x k_y}{\mu_{zz}}-\frac{\omega}{c}\frac{\varepsilon_{xz}\varepsilon_{zy}}{\varepsilon_{zz}} \\ \frac{\omega}{c}\varepsilon_{yx}+\frac{c}{\omega}\frac{k_x k_y}{\mu_{zz}}-\frac{\omega}{c}\frac{\varepsilon_{yz}\varepsilon_{zx}}{\varepsilon_{zz}} & \frac{\omega}{c}\varepsilon_{yy}-\frac{c}{\omega}\frac{k_x^2}{\mu_{zz}}-\frac{\omega}{c}\frac{\varepsilon_{yz}\varepsilon_{zy}}{\varepsilon_{zz}} \end{bmatrix},$$

$$a_{12}=\begin{bmatrix} k_y\left(\frac{\varepsilon_{xz}}{\varepsilon_{zz}}-\frac{\mu_{zx}}{\mu_{zz}}\right) & -k_x\frac{\varepsilon_{xz}}{\varepsilon_{zz}}-k_y\frac{\mu_{zy}}{\mu_{zz}} \\ k_x\frac{\mu_{zx}}{\mu_{zz}}+k_y\frac{\varepsilon_{yz}}{\varepsilon_{zz}} & k_x\left(\frac{\mu_{zy}}{\mu_{zz}}-\frac{\varepsilon_{yz}}{\varepsilon_{zz}}\right) \end{bmatrix},$$

$$a_{21}=\begin{bmatrix} k_y\left(\frac{\varepsilon_{zx}}{\varepsilon_{zz}}-\frac{\mu_{xz}}{\mu_{zz}}\right) & k_x\frac{\mu_{xz}}{\mu_{zz}}+k_y\frac{\varepsilon_{zy}}{\varepsilon_{zz}} \\ -k_x\frac{\varepsilon_{zx}}{\varepsilon_{zz}}-k_y\frac{\mu_{yz}}{\mu_{zz}} & k_x\left(\frac{\mu_{yz}}{\mu_{zz}}-\frac{\varepsilon_{zy}}{\varepsilon_{zz}}\right) \end{bmatrix},\text{and}$$

$$a_{22}=\begin{bmatrix} \frac{\omega}{c}\mu_{xx}-\frac{c}{\omega}\frac{k_y^2}{\varepsilon_{zz}}-\frac{\omega}{c}\frac{\mu_{xz}\mu_{zx}}{\mu_{zz}} & \frac{\omega}{c}\mu_{xy}+\frac{c}{\omega}\frac{k_x k_y}{\varepsilon_{zz}}-\frac{\omega}{c}\frac{\mu_{xz}\mu_{zy}}{\mu_{zz}} \\ \frac{\omega}{c}\mu_{yx}+\frac{c}{\omega}\frac{k_x k_y}{\varepsilon_{zz}}-\frac{\omega}{c}\frac{\mu_{yz}\mu_{zx}}{\mu_{zz}} & \frac{\omega}{c}\mu_{yy}-\frac{c}{\omega}\frac{k_y^2}{\varepsilon_{zz}}-\frac{\omega}{c}\frac{\mu_{yz}\mu_{zy}}{\mu_{zz}} \end{bmatrix}$$

In this work, the anisotropic ambient medium has the following properties that can be given in the following dyadic form

$$\begin{aligned} \overline{\overline{\varepsilon}}_a &= \varepsilon_{xa}\overline{xx}+\varepsilon_{ya}\overline{yy}+\varepsilon_{xa}\overline{zz} \\ \overline{\overline{\mu}}_a &= \mu_{xa}\overline{xx}+\mu_{ya}\overline{yy}+\mu_{xa}\overline{zz} \end{aligned} \tag{B.3}$$

where $\overline{x}, \overline{y}, \text{and}\, \overline{z}$ are the unit vectors along the *x*, *y*, and *z* directions, respectively. These properties provide two real and two imaginary *z*-directional wavenumbers corresponding to propagating and evanescent modes. These wavenumbers are the eigenvalues of the 4×4 matrix iJA in the medium, and the associated eigenvectors are the field modes in that medium.

Appendix C

The magnetoelectric tensors of the pseudochiral omega slab depend on the orientation of the Ω-shaped particles in the host medium. Two reciprocal pseudochiral omega structures are possible: the *xy*-omega (Ω_{xy}) and the *yz*-omega (Ω_{yz}). The *yz*-omega (Ω_{yz}) structure is shown in Fig. 2(a). The permittivity, permeability, and magnetoelectric tensors for this pseudochiral omega medium can be given in the following dyadic form

$$\begin{aligned} \overline{\overline{\varepsilon}}_s &= \varepsilon_{xs}\overline{xx}+\varepsilon_{ys}\overline{yy}+\varepsilon_{zs}\overline{zz} \\ \overline{\overline{\mu}}_s &= \mu_{xs}\overline{xx}+\mu_{xs}\overline{yy}+\mu_{zs}\overline{zz} \\ \overline{\overline{\xi}}_s &= i\Omega\overline{yz} \\ \overline{\overline{\zeta}}_s &= -i\Omega\overline{zy} \end{aligned} \tag{C.1}$$

where the Ω parameter is the coupling coefficient between the electric and magnetic fields along the *y* and *z* directions, respectively. The *xy*-omega (Ω_{xy}) structure is shown in Fig. 2(b). The permittivity, permeability, and magnetoelectric tensors for this pseudochiral omega medium can be given in the following dyadic form

$$\begin{aligned} \overline{\overline{\varepsilon}}_s &= \varepsilon_{xs}\overline{xx}+\varepsilon_{ys}\overline{yy}+\varepsilon_{zs}\overline{zz} \\ \overline{\overline{\mu}}_s &= \mu_{xs}\overline{xx}+\mu_{xs}\overline{yy}+\mu_{zs}\overline{zz} \\ \overline{\overline{\xi}}_s &= i\Omega\,\overline{xy} \\ \overline{\overline{\zeta}}_s &= -i\Omega\overline{yx} \end{aligned} \tag{C.2}$$

where the Ω parameter is the coupling coefficient between electric and magnetic fields along the *x* and *y* directions, respectively.
